# Long-Duration Response to Levodopa in Patients with Early Parkinson’s Disease: Pharmacodynamics, Neuroplasticity, and Clinical Implications

**DOI:** 10.3390/biomedicines14091950

**Published:** 2026-08-30

**Authors:** Giorgia Sciacca

**Affiliations:** Unit of Neurology, Policlinico University Hospital “G. Rodolico-San Marco”, 95123 Catania, Italy; giorgia8sciacca@gmail.com

**Keywords:** Parkinson’s disease, levodopa, long-duration response, drug-naïve, pharmacodynamics, neuroplasticity, motor learning, neurophysiology

## Abstract

**Background:** The most effective pharmacological treatment for Parkinson’s disease (PD) remains levodopa, whose responsiveness is distinguished into two temporally and mechanistically distinct components: the short-duration response (SDR), which lasts hours, closely paralleling drug plasma concentration, and the long-duration response (LDR), which derives from prolonged administration of levodopa and persists for days to weeks after drug discontinuation. The mechanisms underlying the LDR and, in particular, the behavior in early PD patients are incompletely understood. This review aims to gather current evidence on the pharmacodynamics, neurobiological substrate, natural history, and clinical implications of the LDR, with a specific focus on early PD patients. **Methods:** A narrative review was conducted based on a selection of original research articles, clinical studies, and reviews identified through PubMed/MEDLINE, covering the period from January 1995 to July 2026. Studies were selected for their methodological relevance to the characterization of LDR in drug-naive or early-stage PD patients, including pharmacodynamic studies, neuroimaging investigations, and neurophysiological assessments. **Results:** Evidence consistently demonstrates that the LDR is a pharmacodynamically distinct component of levodopa response, present from the earliest stages of dopaminergic therapy. In drug-naive patients, the LDR represents a quantitatively substantial fraction of total levodopa benefit, and it is linked to modifications in cortical excitability, dopaminergic neuroplasticity, and motor learning. Structural neuroimaging identifies predisposing gray matter features in motor cortex regions. Whether LDR magnitude declines with disease and treatment duration, and thereby underlies the emergence of motor fluctuations, remains disputed across studies. This discrepancy appears attributable, at least in part, to differences in how the LDR is measured (direct washout assessment versus inference against a projected untreated trajectory) rather than necessarily to divergent underlying biology. **Conclusions:** The LDR to levodopa represents a neurobiologically and clinically significant dimension of levodopa response. Its characterization in early PD patients may refine therapeutic strategies, inform the design of neuroprotection trials, and open new possibilities for precision medicine approaches.

## 1. Introduction

Levodopa has remained the cornerstone of symptomatic pharmacotherapy for Parkinson’s disease (PD) for over five decades. Its introduction in the late 1960s transformed PD management by directly replenishing the striatal dopamine deficit caused by nigrostriatal degeneration [1], and levodopa response represents both a diagnostic criterion and a prognostic marker in clinical practice. In the earliest pharmacokinetic studies of the drug, the therapeutic response was noted not to track plasma levodopa concentrations in a simple manner [2]. The motor benefit derived from chronic dopaminergic therapy substantially exceeded what could be expected from transient plasma drug elevations [3], a phenomenon formally characterized by Nutt and colleagues [4], prompting the distinction between two pharmacodynamically discrete components of levodopa response [4,5]: the short-duration response (SDR) and the long-duration response (LDR). The SDR to levodopa is an improvement lasting hours following a single dose of levodopa and matching plasma drug concentrations [4]. The LDR is defined as a sustained clinical benefit that accumulates over repeated dosing and persists for days to weeks after drug discontinuation [4]. The two components are pharmacodynamically distinct and interact: the prevailing LDR modulates the threshold and magnitude of the superimposed SDR [6,7]. Because its temporal profile renders it invisible during stable therapy, the LDR has historically attracted less attention than the SDR. Nevertheless, evidence indicates that the LDR to levodopa represents a distinct component of total levodopa benefit, relevant to disease progression, neurobiology of dopamine replacement [8] and the controversial relationship with motor complications [7,9,10,11]. This review focuses specifically on the LDR to levodopa in drug-naive PD patients, a population in whom first-ever levodopa exposure allows its observation in the purest form, unconfounded by prior treatment or established fluctuations. A comprehensive synthesis of current knowledge on the definition, mechanisms, clinical characterization, neuroimaging analyses, neurophysiological assessments, and therapeutic implications of LDR in early PD is here provided.

## 2. Materials and Methods

A targeted PubMed/MEDLINE search (January 1995–July 2026), reflecting the most recent literature available at the time of this revision, including two items published in 2026, combined the following terms “levodopa” OR “L-DOPA” AND “long-duration response” OR “LDR” AND “Parkinson’s disease” OR “Parkinson disease”, cross-referenced with terms for drug-naive/early-stage disease, pharmacodynamic, neuroplasticity, neuroimaging, and neurophysiology. The 1995 lower bound has been chosen to capture the pharmacodynamic literature that formally distinguishes the SDR and LDR to levodopa using standardized testing (e.g., the subacute levodopa test). Pre-1995, foundational studies that first described the phenomenon (1971–1992) were identified through backward reference-list screening of the key papers above and are included where directly relevant [2,3]. Reference lists of the key articles were also screened to identify additional foundational sources. Studies were selected for their specific relevance to the LDR characterization in drug-naive or early PD patients, based on title and abstract screening. Duplicates, articles lacking patient data, and case reports were excluded, though their reference lists were reviewed for additional relevant literature. Screening was conducted by a single author, which may have limited the findings due to possible selection bias. Given the heterogeneity of study designs, ranging from acute motor response challenges to structural MRI and electrophysiological measures, and the limited number of studies focusing specifically on drug-naive or early PD patients, a systematic review or meta-analysis was not methodologically appropriate. Indeed, the aforementioned research showed 68 studies, of which only 12 were focused strictly on the topic. Two of the studies discussed in this review are primary data papers from the author’s own research group [12,13]; it was held to the same critical standard as the wider literature, representing a further potential source of bias. For all these reasons, evidence was qualitatively synthesized and organized into thematic domains in a narrative review structure.

## 3. Results

### 3.1. Natural History and Clinical Evidence

The study of LDR in drug-naive patients offers a unique scientific opportunity: the absence of prior dopaminergic treatment allows characterization of LDR emergence without the confounding influence of chronic medication-induced neuroadaptations and established motor fluctuations. Such a population is, however, difficult to access in settings where PD is promptly diagnosed and treated. In sub-Saharan Africa, where levodopa access has historically been limited, a cohort of 30 patients (age at onset 58 ± 14 years, disease duration 7 ± 4 years) starting levodopa after several years of untreated disease was followed prospectively for two years with serial ON/OFF assessments [10]. First-ever exposure produced substantial, sustained improvement in OFF-state motor scores, and modelling against the expected untreated trajectory suggested that levodopa reduced the annual decline in OFF-state scores, a finding with implications for distinguishing potentially disease-modifying effects from persistence of LDR into the OFF-state assessment window itself [10]. Using this same notional-untreated-trajectory approach, the magnitude of the LDR itself was estimated at 60–65% of total motor benefit, independently of disease duration, and a sustained benefit was still detectable at 4 years of follow-up even though two-thirds of the cohort had already developed diurnal motor fluctuations by 1 year [10]. The LDR in early-treated PD patients is not only detectable, but its achievement is also modulated by the dosing regimen used to induce it. In a double-blind, crossover trial comparing three 15-day levodopa regimens in 24 patients with early PD, a sustained LDR was achieved by all patients receiving 250 mg every 24 h or 250 mg every 8 h but by only 17% of patients receiving 125 mg every 8 h, indicating that the achievement of a sustained LDR depends on the size of the individual dose rather than on total daily dose alone, and that regimens of small, divided doses may be a questionable strategy for inducing a sustained LDR [14]. Once induced, the LDR reaches full development within days to weeks of treatment initiation [14]. Estimates indicate that it may represent 30–50% in early PD using pharmacodynamic modelling of longitudinal treatment response over the first year and first four years of therapy [9,15,16]. These findings have been further examined at the population level in the PPMI cohort, one of the largest multicenter studies of drug-naive PD in which a mixed linear model derived from 245 untreated participants was used to predict expected untreated motor trajectories, against which the LDR to levodopa was then calculated in 148 initially therapy-naive participants [11]. Among the 98 participants with an observable LDR, its magnitude accounted for half of the total levodopa response, confirming the prevalence of LDR in a geographically diverse sample and supporting its use as a standard pharmacodynamic outcome [11]. The 50 PD patients without an observable LDR progressed faster across several motor and non-motor domains than those with an observable LDR [11].

### 3.2. Neurobiological Mechanisms: Dopaminergic Neuroplasticity as the Substrate of the LDR to Levodopa

Dopamine is thought to act not as a fast neurotransmitter modulating the immediate activity of striatal projection neurons but as a key regulator of synaptic plasticity in cortico-striatal circuits, specifically long-term potentiation (LTP) and long-term depression (LTD) at glutamatergic synapses on medium spiny neurons [8]. D1-mediated signaling in the direct pathway facilitates LTP, while D2-mediated signaling in the indirect pathway facilitates LTD [8]; progressive dopamine depletion disrupts this balance, favoring aberrant plasticity that underlies both motor symptoms and maladaptive motor learning before treatment [8]. Exogenous dopamine replacement via levodopa partially restores the capacity for experience-dependent synaptic plasticity, and LDR, within this framework, represents the time-integrated consequence of this restored plasticity across repeated doses [8]. Early initiation of dopamine replacement therapy may be especially important for preserving neuroplasticity capacity, as prolonged dopamine depletion may engender irreversible maladaptive synaptic changes that limit subsequent plasticity [8]. This perspective provides a neuroscientific rationale for the clinical observation that LDR is most prominent and therapeutically most significant in drug-naive PD, a period during which the system retains the greatest capacity for exploiting dopaminergic restoration for neuroplasticity benefit. The same cortico-striatal plasticity framework proposed to underlie the beneficial LDR has also been implicated in the opposite, treatment-limiting outcome of chronic dopaminergic stimulation: levodopa-induced dyskinesia [17]. In animal models, chronic levodopa treatment restores cortico-striatal LTP in both dyskinetic and non-dyskinetic parkinsonian rats, but only non-dyskinetic animals retain the ability to subsequently de-potentiate these synapses; dyskinetic animals show a selective loss of this bidirectional (LTP/LTD) plasticity [17]. This suggests that adaptive plasticity (potentially underlying the LDR) and maladaptive plasticity (underlying dyskinesia) may represent two divergent outcomes of the same dopamine-dependent cortico-striatal mechanism [8,17], raising the open question of whether individual variability in LDR expression and susceptibility to dyskinesia are mechanistically related, inversely related, or largely independent. Whether a highly robust LDR initially protects against or predisposes to these motor complications as the disease advances in drug-naive PD represents an important target for future longitudinal investigation.

### 3.3. Pharmacodynamics of the LDR to Levodopa

The pharmacodynamics of levodopa response in PD should not be reduced to a simple plasma concentration-effect relationship. The SDR constitutes the clinically most visible component: it emerges within minutes to hours of oral levodopa administration, peaks in parallel with plasma drug concentration, and dissipates as plasma levels decline [18]. This reflects the rapid conversion of exogenous levodopa to dopamine within surviving presynaptic terminals and the subsequent activation of postsynaptic dopamine receptors in the striatum. The LDR, conversely, does not follow peripheral pharmacokinetic fluctuations: it builds over days of repeated dosing, and its magnitude reflects cumulative dopaminergic stimulation rather than individual dose levels [18]. Detailed pharmacodynamic modelling of the LDR was performed, demonstrating that it follows a saturable dose-accumulation function that reaches a plateau under steady-state dosing conditions, and declines exponentially during washout with a time constant markedly exceeding the elimination half-life of levodopa [18]. A pharmacokinetic-pharmacodynamic modelling study, comparing 13 de novo and 12 chronically treated PD patients given 2-h intravenous levodopa infusions on two occasions 3 days apart, found that a three-compartment model (fast equilibration, slow equilibration, and dopa synthesis) best described the data [19]. This directly implies that the LDR itself is not a single kinetic entity but comprises components with distinct equilibration time constants [19]. In the chronically treated group, partial loss of the LDR during the 3-day levodopa-free interval reduced baseline tapping speed and increased the peak levodopa-induced response, directly demonstrating that even a relatively short, 3-day washout captures only a partial, not complete, loss of the LDR [19]. Independent evidence that the LDR reflects a genuine postsynaptic pharmacodynamic phenomenon, rather than persisting presynaptic dopamine storage or metabolism, comes from a study in which daytime infusions of apomorphine, as a direct postsynaptic D1/D2 agonist that bypasses presynaptic levodopa-to-dopamine conversion entirely, sustained the LDR in 7 PD patients with fluctuating PD from whom levodopa itself had been withdrawn for 3 days [20].

The interaction between SDR and LDR has important clinical implications. The prevailing LDR state at any given time acts as a tonic background upon which the phasic SDR is superimposed [6]. High LDR, as observed in early PD disease and at the initiation of therapy, provides a stable motor baseline that smooths dose-related fluctuations and ensures consistent motor control throughout the day. Conversely, the reduction of the LDR, which occurs as disease progresses and dopaminergic denervation advances, leads to increased dependence on individual SDR cycles and greater motor variability [6,7]. It should be noted, however, that this classical formulation, in which LDR reduction is what drives greater SDR-related variability, is itself one side of the controversy examined in Section 3.7.

### 3.4. Methodological Approaches to the LDR Lo Levodopa Assessment

The LDR assessment requires structured washout protocols, often poorly tolerated in advanced disease. The subacute levodopa test addresses this by appending a brief standardized off-drug window to regular dosing, for LDR estimation [21]. Longitudinal designs in drug-naive PD populations, as in the sub-Saharan African cohort, instead track OFF-state function over time [10], while large cohort studies such as PPMI have used mixed linear models across hundreds of untreated patients to estimate the LDR prevalence and determinants [11]. Each approach carries specific strengths and limitations: the subacute test provides precise pharmacodynamics data but is labor-intensive; natural history designs in drug-naive populations are powerful but logistically demanding; large cohort analyses offer generalizability but then rely on proxies rather than direct LDR measurement. This methodological heterogeneity is not incidental: it corresponds to two fundamentally different ways of operationalizing the LDR, and it is central to understanding why the literature on LDR trajectory over time is divided. One approach, exemplified by the subacute levodopa test [21] and the 1-year follow-up study of LDR loss [7], measures the LDR directly, by comparing motor status after a structured washout period to motor status on treatment. The other approach, used by the sub-Saharan African cohort [10] and the PPMI-cohort analysis [11], infers the LDR indirectly, by comparing observed OFF-state motor scores on treatment to a modelled (“notional”) trajectory of what motor disability would have been had the patient never been treated. Each approach carries assumptions that could plausibly generate divergent conclusions about whether the LDR erodes over time, independently of any true underlying biological difference. First, the notional-trajectory approach requires extrapolating a projected untreated disease course over years, which implicitly assumes that motor decline follows a stable (typically linear) trajectory. This assumption is not firmly established, since motor score trajectories in PD have been reported to be non-linear over the disease course, which could bias the projected comparator in ways that are difficult to predict or correct for. Second, both direct and indirect approaches typically rely on an overnight or “practically defined” OFF state (approximately 10–12 h after the last dose) as their reference point. Because the LDR decays with a time constant on the order of days rather than hours [18], a substantial fraction of the LDR is likely still present at this time point, meaning that neither the OFF state used as a treated measurement nor, where applicable, the pre-treatment baseline used to anchor the notional trajectory, may be fully free of LDR contamination. A further methodological axis compounds this picture: while some pharmacodynamic studies have relied on tapping speed as a continuous index of bradykinesia and consequently of LDR magnitude [9,15,19], natural-history and large-cohort studies have relied on motor multi-item ordinal clinical scales with different sensitivity and floor/ceiling behavior [10,11]. The extent to which these two outcome types are quantitatively comparable for estimating LDR magnitude or decay has not, to our knowledge, been directly established. Thus, divergent conclusions reached by studies measuring LDR to levodopa may reflect, at least in part, these methodological differences rather than necessarily representing contradictory biological findings—an interpretation that could reconcile the two bodies of evidence.

### 3.5. Neurophysiological Correlates of the LDR to Levodopa and Motor Learning

Evidence that the LDR to levodopa could be a manifestation of “rescued” motor learning was consistent with data-mining of the ELLDOPA trial, showing that the dominant (more active) hand developed a significantly greater practically defined OFF (predose) improvement in finger-tapping than the non-dominant hand over 42 weeks of levodopa treatment, in a dose-dependent fashion [22]. These findings revealed that activity-dependent, dopamine-facilitated motor learning contributed to the LDR [22]. On these grounds, the neuroplasticity framework of LDR to levodopa in early PD patients was recently examined through neurophysiological tests, combining indirect markers of neuroplasticity with motor learning activities [12]. A prospective study was conducted in 41 drug-naive PD patients (and 24 age- and sex-matched healthy controls) initiating levodopa therapy (15-day treatment with levodopa/carbidopa 250/25 mg daily), systematically examining relationships between LDR achievement, motor learning performance, and neurophysiological markers of cortical plasticity [12]. Patients were stratified into four groups by the 2 × 2 combination of LDR achievement (LDR+/LDR-) and assignment to a learning motor exercise (LME) or no motor exercise (NME), with no differences in clinical or neurophysiological parameters at baseline, and were assessed at the starting point and following a structured protocol designed to dissociate LDR from SDR [12]. Neurophysiological measurements were obtained using surrogate markers of neuroplasticity, including motor evoked potentials (MEPs), P300 event-related potentials, and Bereitschaftspotential (BP), established indices of corticospinal excitability, cognitive-attentional demands processing in early motor learning phases, and motor readiness, respectively [12]. MEPs were included even if primarily indices of corticospinal excitability rather than genuine LTP-like plasticity markers, as in combination with the other neurophysiological scores of motor learning. The findings revealed that patients who achieved a sustained LDR to levodopa and underwent the motor learning protocol (the LDR+/LME group specifically) demonstrated significant improvements in neurophysiological parameters (reduced latencies and increased amplitude for MEPs; reduced latencies for both P300 and BP), reflecting changes in cortical excitability, cognitive-attention domains and motor preparation networks that outlasted the pharmacokinetic window of individual doses [12]. These findings pose the LDR as a neurobiologically active state that potentiates the brain’s capacity for motor learning-driven neuroplasticity, with direct relevance for motor rehabilitation strategies at the early stage of disease [12]. In fact, drug-naïve patients retain a degree of striatal and cortical integrity that renders motor circuits more responsive to plasticity-promoting stimuli. To elaborate, the LDR to levodopa represents an optimal pharmacological state for the facilitation of rehabilitation-driven neuroplasticity, and the timing of motor learning interventions relative to the LDR phase may critically influence therapeutic outcomes [8,12].

### 3.6. Structural Neuroimaging Correlates

Evidence from structural neuroimaging has begun to identify brain features that determine individual variability in the LDR to levodopa magnitude and achievement. An exploratory voxel-based morphometry (VBM) study was conducted in a cohort of 24 drug-naive PD patients who underwent brain MRI prior to levodopa initiation and were subsequently stratified according to whether they achieved a sustained LDR to levodopa [13]. The analysis revealed significant differences in gray matter volume between LDR achievers and non-achievers in the precentral gyrus and middle frontal gyrus, cortical regions belonging to the primary motor and premotor networks [13]. These differences were present before any dopaminergic treatment, indicating that structural features of the motor cortex, rather than being a consequence of levodopa exposure, may act as determinants of individual capacity to develop LDR [13]. This is consistent with the neuroplasticity framework: integrity of the premotor and primary motor cortex is a prerequisite for experience-dependent plasticity in cortico-striatal circuits [8]. Patients with greater gray matter preservation in these regions may retain a superior capacity for dopamine-driven cortico-striatal LTP, providing the structural basis for more robust LDR development [8,13]. The exploratory nature of the reported study, with its relatively small sample size and cross-sectional MRI design, limits definitive conclusions. Moreover, to our knowledge, it remains the only published neuroimaging study to address this specific question in the drug-naïve PD population; independent replication in a larger, ideally multicenter cohort is needed before these structural findings can be considered established LDR predictors. Nevertheless, the identification of pre-treatment structural predictors of LDR opens an important path for the development of imaging biomarkers capable of informing personalized therapeutic strategies [13].

### 3.7. The LDR to Levodopa over Time: An Open Controversy, with Implications for Disease Progression and Motor Complications

Although the primary focus of this review is the behavior of LDR to levodopa in early PD patients, fully understanding the natural history of LDR necessitates an explicit extension into LDR longer-term evolution as disease and treatment duration progress, even though it will not be the subject of extensive coverage. The pathophysiology of motor fluctuations in PD more broadly has been the focus of fundamental, prior studies [23]. An early longitudinal study, directly measuring the LDR by comparing motor status before and after a structured washout in the same patients on two occasions approximately one year apart, found that the LDR declined over the disease course [7]. Twenty-four per cent of the 17 patients studied lost a previously demonstrable LDR over the 1-year interval, and this loss was associated with the clinical emergence of predictable wearing-off [7]. As tonic LDR support declines, SDR-driven motor oscillations increase in amplitude and motor control becomes more sensitive to dose timing [6,7], such that wearing-off could reflect a shift in the pharmacodynamic architecture of levodopa response rather than a purely pharmacokinetic phenomenon [7]. This erosion model, however, is directly challenged by evidence from two larger and longer-term cohorts that inferred the LDR against a projected untreated trajectory rather than measuring it directly. In the sub-Saharan African cohort, the estimated LDR magnitude did not differ significantly across patients at the various stages of the disease, and a substantial LDR remained detectable at 4 years of treatment even though two-thirds of the cohort had already developed diurnal motor fluctuations by 1 year [10]. These findings indicated that, in this cohort, the onset of fluctuations did not require LDR to have been lost [10]. In the PPMI-cohort analysis, no significant change in LDR magnitude was observed over follow-up periods of up to 10 years, and the LDR magnitude was not associated with the onset of motor complications [11]. On the other hand, the complete absence of an observable LDR, rather than its gradual decline, was associated with faster disease progression across several motor and non-motor domains [11]. The direct measurement of LDR and SDR, not by inference, in the same 18 PD patients at baseline and after 6, 12, 24, and 48 months of levodopa therapy, using both overnight and 3-day withdrawal at each visit, showed different findings [9]. When measured after overnight withdrawal only, neither the SDR nor the LDR changed detectably over the 4 years [9]. However, when measured after 3 days of withdrawal in the same patients, SDR magnitude increased progressively, and LDR decayed more rapidly, detecting a form of LDR erosion [9]. Critically, the SDR magnitude at 4 years was inversely related to the LDR magnitude, and reported motor fluctuations tended to be associated with a large SDR rather than a small one [9]. The interpretation was that a larger, not a shortened, LDR is more important to the development of fluctuations, by elevating the practical-OFF baseline against which the SDR is superimposed, and that improving practical-OFF motor function specifically (rather than simply preserving a large LDR) may be a viable strategy to treat fluctuations [9]. This is a genuinely different claim from both positions above: it does not simply assert that the LDR is stable (as in [10,11]), nor that its loss drives fluctuations (as in [7]), but that its evolution is washout-duration-dependent and that, if anything, a larger LDR is the more important correlate of fluctuations [9]. These four studies [7,9,10,11] therefore reach materially different conclusions regarding the direction of its relationship to motor complications where one is found at all. In the case of the PPMI-cohort [11] remains to be clarified whether LDR decline is mechanistically linked to motor complications at all—a tension explicitly noted in the editorial accompanying the PPMI-cohort report, which likewise frames the trajectory of the LDR over time as unresolved [24], consistent with an earlier call for the field to reconsider oversimplified models of levodopa response [25]. To elaborate, the most defensible position at present is that this represents a genuine and unresolved controversy in the field, which may be attributable at least in part to the differing methodologies used to estimate LDR (direct washout measurement in a small, single-center cohort versus large-cohort inference against a projected untreated trajectory, washout duration, and outcome measure—tapping speed versus motor scale) [9,19], rather than necessarily reflecting divergent underlying biology. To the best of our knowledge, no studies have applied all of these methodological dimensions in combination to the same cohort, which would be needed to directly adjudicate between these possibilities. Until such data are available, the relationship between LDR trajectory and disease/treatment duration should be treated as an open question rather than an established fact, and the mechanistic hypotheses below are presented as applicable specifically to the erosion model, pending resolution of this methodological divergence. Should genuine LDR erosion occur in at least a subset of patients, several non-mutually-exclusive mechanisms have been proposed to explain it: progressive nigrostriatal denervation reducing buffering capacity and postsynaptic signaling [7], altered dopamine receptor expression/sensitivity and receptor desensitization from chronic pulsatile stimulation further degrading the postsynaptic substrate [8], and a possible shift from adaptive toward maladaptive cortico-striatal plasticity [17]. However, these mechanisms remain hypothetical explanations for a phenomenon whose existence at a population level is currently disputed by the evidence summarized above.

## 4. Discussion

**Figure 1:**
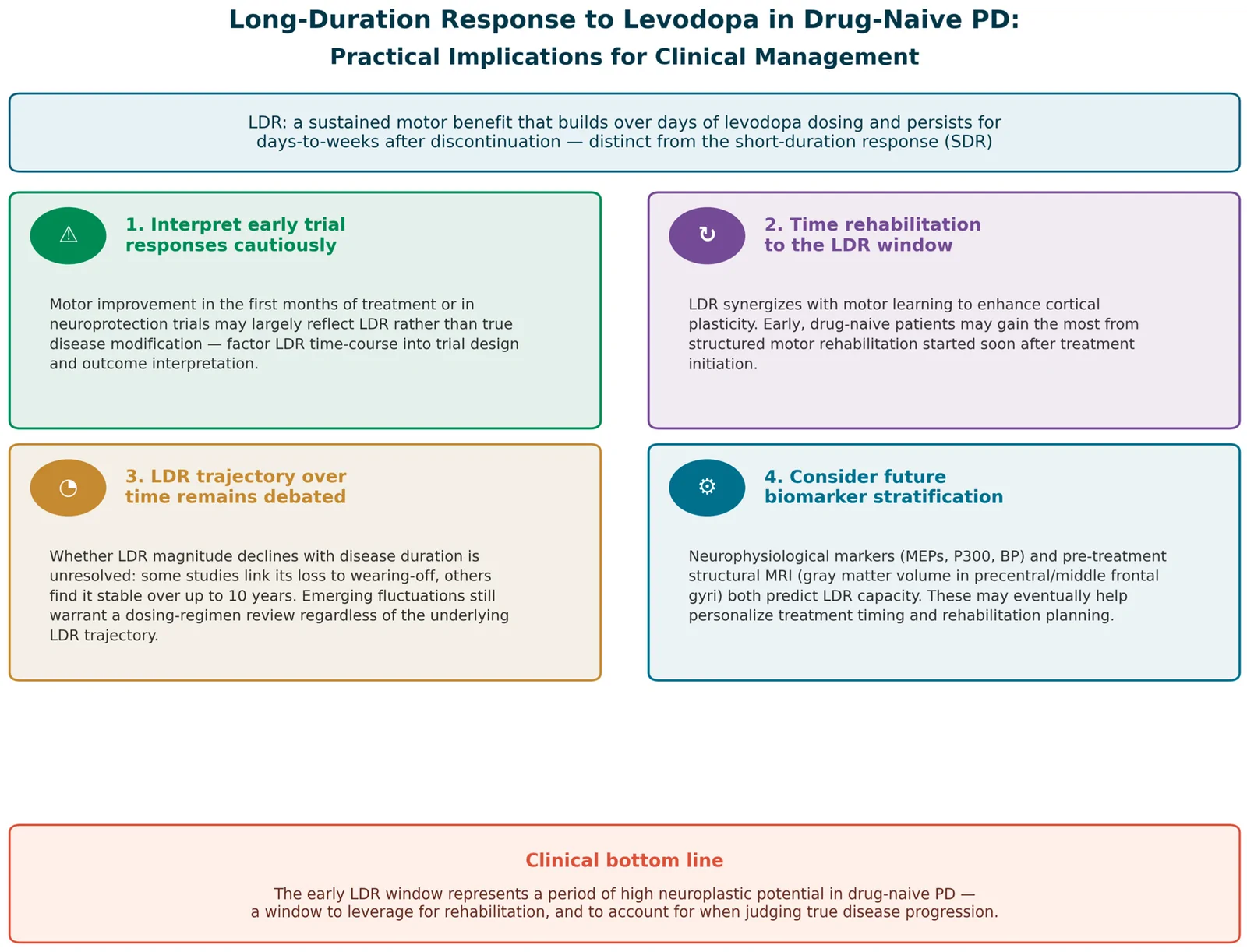
Practical implications for clinical management of drug-naïve patients affected by Parkinson’s disease. The figure summarizes four practical implications of the evidence reviewed, organized around a central definition of the LDR (top band). Box 1 addresses the risk of overestimating disease-modifying effects in early treatment or neuroprotection trials if the LDR contribution is not accounted for (Section 4). Box 2 addresses the proposed synergy between LDR and motor learning, supporting early, structured motor rehabilitation (Section 3.5). Box 3 shows that the trajectory of LDR magnitude over the disease course is an open, actively debated question rather than an established progressive decline (Section 3.7). Box 4 addresses candidate neurophysiological and structural predictors of LDR capacity that may eventually support individualized treatment planning (Section 3.5 and Section 3.6). The bottom panel summarizes the overall clinical message. Abbreviations: PD: Parkinson’s disease; LDR: long-duration response to levodopa; SDR: short-duration response to levodopa; MEPs: motor evoked potentials; BP: Bereitschaftspotential.

The characterization of the LDR to levodopa in early PD patients carries several important implications for clinical practice and research (summarized in Figure 1, which depicts the LDR clinical predominance and neuroplastic activity in early disease, resulting in the practical implications discussed in this section). First, the predominance of LDR in the early stages of disease means that assessments of treatment efficacy performed within the first months of therapy may substantially overestimate the contribution of SDR, if LDR is not properly accounted for [9,16]. This has particular relevance for neuroprotection trials conducted in drug-naive or recently treated patients, where observed motor improvement may reflect LDR rather than true disease modification [10]. The sub-Saharan African cohort illustrates this directly: apparent slowing of motor disability progression should be weighed against the possibility that a sustained LDR persisted into the OFF-state assessment window itself [10]. The distinction between true neuroprotection and LDR-mediated effects requires study designs that explicitly model the time course and magnitude of LDR. This consideration should be incorporated into the design of future neuroprotective trials. Second, the identification of neurophysiological and structural predictors of LDR in drug-naive patients [12,13], despite the discussed caveats, opens the prospect of individualized LDR assessment as part of therapeutic planning. Patients predicted to achieve a robust LDR may be particularly amenable to motor rehabilitation strategies designed to exploit the synergy between LDR and motor learning [12], while patients with anticipated poor LDR may benefit from earlier introduction of continuous dopaminergic stimulation strategies. Third, in the subset of patients in whom genuine LDR loss occurs—a premise that, as detailed in Section 3.7, is itself disputed rather than settled— strategies aimed at preserving or restoring LDR, including modified-release levodopa formulations, dopamine agonists, or device-aided continuous drug delivery, may protect the neuroplasticity substrate itself, not only through pharmacokinetic optimization [7,10,12]. This is presented as a hypothesis rather than an established therapeutic principle. The STRIDE-PD trial, which randomized 747 early PD patients to initiate levodopa/carbidopa with or without the COMT inhibitor entacapone specifically to test whether more continuous levodopa delivery would delay motor complications, found different findings [26]. Indeed, the more continuously delivered combination arm had a significantly shorter time to onset of dyskinesia and a higher frequency of dyskinesia by the end of the study than standard levodopa/carbidopa [26]. This counter-evidence does not rule out that LDR-preserving strategies specifically (as opposed to continuous dopaminergic stimulation generally, which acts through additional and only partly overlapping mechanisms) could still be beneficial, and that this hypothesis warrants direct testing in prospective clinical trials in drug-naive patients. Large cohorts such as PPMI [11] already provide an epidemiological foundation for biomarker-driven stratification, linking clinical phenotype to molecular and imaging markers of dopaminergic integrity within a precision medicine framework in which LDR magnitude could be monitored as a pharmacodynamic endpoint alongside conventional clinical measures. A further confound is represented by the placebo response in PD. It was observed that it is itself dopaminergic, with PET studies using [11C] raclopride showing that placebo administration triggers substantial endogenous striatal dopamine release mediated by expectation of clinical benefit, an effect reported to persist for weeks in some trial settings [27]. Because this represents a genuine form of endogenous dopaminergic stimulation, it is not separable, on current evidence, from a sustained OFF-state benefit attributed to the LDR to exogenous levodopa [26]. This possibility has not been addressed by any of the washout-based or notional-trajectory-based studies discussed above. Relatedly, repeated performance of the same motor task across a study can itself produce practice- or test-retest-related improvement independent of any LDR-specific mechanism, a recognized general confound for repeated-measures motor testing (including tapping-speed and motor learning exercises). Finally, the potential contamination of OFF-state measurements by residual LDR has practical implications beyond trial design. The acute levodopa challenge test commonly used to select candidates for device-aided therapies such as deep brain stimulation, and the analogous “levodopa responsiveness” item within the MDS clinical diagnostic criteria for PD [28], are typically interpreted as isolating the SDR, although they still carry a substantial LDR component. The challenge may therefore be to measure the SDR against an LDR-contaminated rather than an LDR-free baseline, a consideration that, to our knowledge, is not routinely addressed in protocols using this test. Despite no specific wearable-based method having yet been validated against a washout-based LDR reference standard, emerging tools could offer a potential path for LDR quantification in both research and clinical settings. Further longitudinal studies are necessary to resolve the controversial aspects of the LDR to levodopa, especially in drug-naïve PD patients.

## 5. Conclusions

The LDR to levodopa in early PD patients is a pharmacodynamically and neurobiologically distinct phenomenon that constitutes a major component of total levodopa benefit at disease onset. The mechanistic underpinning, rooted in dopamine-dependent cortico-striatal plasticity and the capacity to potentiate motor learning, situates the LDR to levodopa at the intersection of pharmacology, neuroscience, and clinical management. Whether its progressive loss with disease advancement provides a neurobiological explanation for the emergence of motor fluctuations, as classically proposed, or whether its apparent stability or erosion is itself an artifact of washout duration and outcome measure, remains an open and actively debated question; resolving it should be a research priority, since it directly bears on whether LDR preservation is a meaningful therapeutic objective in the way classically proposed.

Several other important aspects remain open. The molecular events at the striatal postsynaptic membrane that specifically initiate and maintain the LDR to levodopa state require further elucidation, including the relative contributions of D1 and D2 receptor-mediated plasticity, signaling pathways, and receptor modifications. Whether LDR magnitude at treatment initiation constitutes a reliable prognostic marker for long-term motor outcome and the rate of wearing-off development remains incompletely addressed and constitutes a priority for prospective research, ideally using a single, consistent methodology applied longitudinally within the same cohort to directly resolve the controversy identified. The potential for the LDR to levodopa to be pharmacologically enhanced through optimized dosing schedules, adjunctive agents, or structured motor rehabilitation protocols represents a therapeutically important direction. Overall, the LDR to levodopa represents an important dimension of levodopa treatment, whose study in drug-naive PD patients has enriched our understanding of the early neurobiological consequences of dopamine restoration, with continued research likely to yield insights for disease management and therapeutic innovation.

## Data Availability

No new data were created or analyzed in this study. Data sharing is not applicable to this article.
